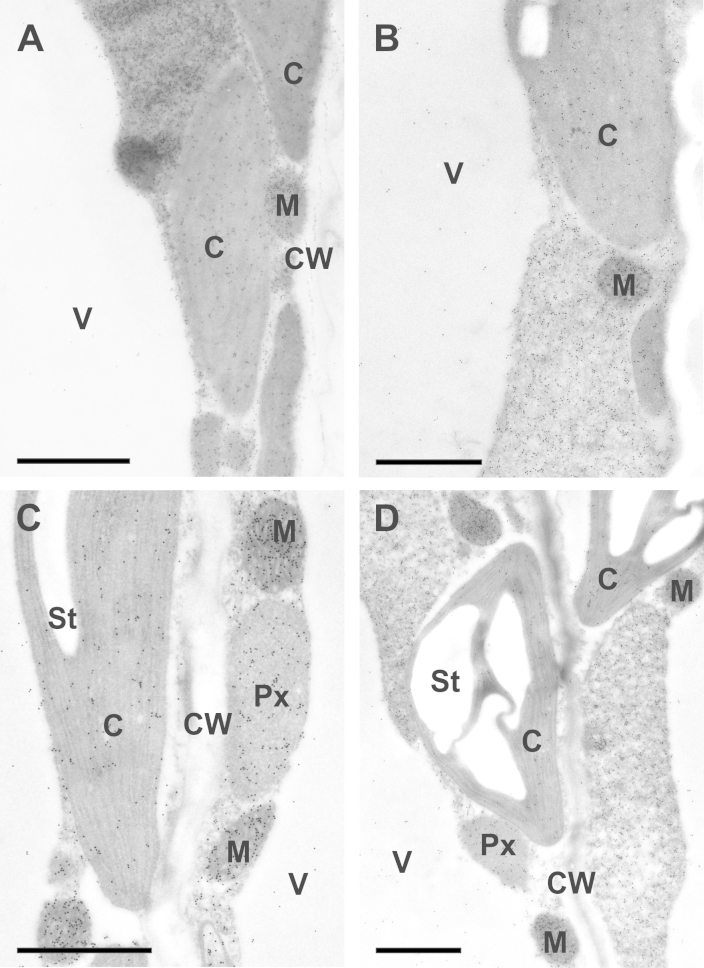# Compartment specific response of antioxidants to drought stress in Arabidopsis

**DOI:** 10.1016/j.plantsci.2014.08.002

**Published:** 2014-10

**Authors:** Barbara Eva Koffler, Nora Luschin-Ebengreuth, Edith Stabentheiner, Maria Müller, Bernd Zechmann

**Affiliations:** a University of Graz, Institute of Plant Sciences, Schubertstrasse 51, 8010 Graz, Austria; b Baylor University, Center for Microscopy and Imaging, One Bear Place #97046, Waco, TX 76798, USA

**Keywords:** Arabidopsis, Ascorbate, Glutathione, Reactive oxygen species, Vacuoles

## Abstract

Compartment specific changes in ascorbate and glutathione contents were studied during drought stress in *Arabidopsis thaliana* Col-0 and in ascorbate and glutathione deficient mutants *vtc2-1* and *pad2-1*, respectively, over a time period of 10 days.

The results of this study revealed a strong decrease of glutathione contents in both mutants (up to 52% in mitochondria of *pad2-1* and 40% in nuclei of *vtc2-1*) at early time points when drought stress was not yet measurable in leaves even though the soil showed a drop in relative water contents. These results indicate that glutathione is used at early time points to signal drought stress from roots to leaves. Such roles could not be confirmed for ascorbate which remained unchanged in most cell compartments until very late stages of drought. During advanced drought stress the strong depletion of ascorbate and glutathione in chloroplasts (up to 50% in Col-0 and *vtc2-1*) and peroxisomes (up to 56% in Col-0) could be correlated with a strong accumulation of H_2_O_2_. The strong increase of H_2_O_2_ and ascorbate in vacuoles (up to 111%) in wildtype plants indicates that ascorbate plays an important role for the detoxification of ROS in vacuoles during drought stress.

## Highlights

• Subcellular H_2_O_2_, ascorbate and glutathione was monitored during drought.
• Glutathione seems to be involved in signaling drought stress from roots to leaves.
• Ascorbate and glutathione decreased whereas H_2_O_2_ accumulated during drought.
• During drought H_2_O_2_ leaked into vacuoles where it was detoxified by ascorbate.
• The breakdown of the antioxidative system during drought favored ROS accumulation.

## Graphical abstract

**Figure:**
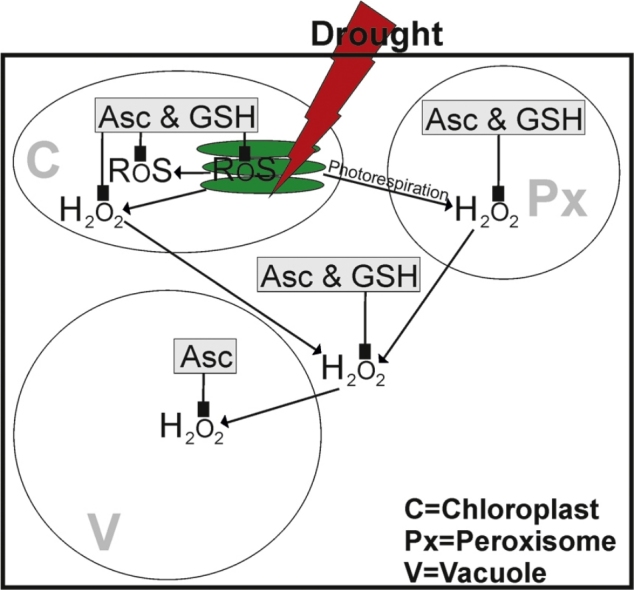

## 1 Introduction

Drought stress in plants is characterized by the continuous loss of water through transpiration and evaporation into the atmosphere while the water uptake is decreased due to reduced water content in the soil. One of the first responses of plants to drought is the closure of stomata which will limit the gas exchange between the leaves and the atmosphere and will decrease the ratio of CO_2_ to O_2_ [1], [2]. This will lead to oxidative stress in illuminated chloroplasts as the lack of CO_2_ in chloroplasts will induce malfunctions of the Calvin cycle which will lead to the exhaustion of the primary electron acceptor NADP and to the block of the electron transport to NADP. Subsequently electrons will be transferred to O_2_ leading to the formation of reactive oxygen species (ROS) [1], [2], [3], [4], [5]. High O_2_ and low CO_2_ levels during drought in plants will additionally favor a process called photorespiration, which leads to the formation of phosphoglycolate. The degradation of this toxic component leads to the production of H_2_O_2_ in peroxisomes [1], [2], [3], [4]. Thus, depending on the degree of water deficiency in plants large amounts of ROS can be produced. As ROS induce lipid peroxidation and oxidative damages of proteins and DNA [6], [7], [8] they have to be detoxified in order to avoid negative effects on plant growth of development. The key players of ROS detoxification during abiotic stress conditions in plants are antioxidants and related enzymes [1], [2], [4], [9], [10]. Among them ascorbate and glutathione have unique roles as they are water soluble and occur in all cell compartments [11], [12] and are therefore used by plants for ROS detoxification throughout the cell as well as signaling purposes between different cell compartments to activate plant defense [3], [4], [5]. As reducing agents they can eliminate ROS individually or through the ascorbate glutathione cycle [3]. Thus it is not surprising that changes in ascorbate and glutathione contents are a commonly observed stress response of plants during drought [2], [10]. In Arabidopsis wildtype plants both glutathione and ascorbate contents were strongly increased during drought stress within the first 72 h [13]. In contrast decreased levels of ascorbate were found in the ascorbate deficient *vtc1* mutants when exposed to drought [13], [14]. Glutathione contents were strongly increased in *vtc1* mutants during drought stress [13]. In another study glutathione contents did not differ between well watered controls and drought stressed Arabidopsis wildtype plants and *vtc-2* mutants whereas ascorbate contents strongly increased in these plants during drought [15]. In tobacco plants unchanged ascorbate and glutathione contents were found during drought [16] whereas in *Agropyron cristatum* leaves a strong increase of glutathione, ascorbate and related enzymes was detected after drought [17].

The above mentioned responses of antioxidants in plants to drought have mainly been investigated by using biochemical methods in whole leaves or organs. Nevertheless, investigations about the situation in whole leaves do not reflect the situation in single cells and organelles. This is especially critical during drought stress as it induces the formation of ROS primarily in chloroplasts and peroxisomes and as it is unclear how and if ROS spread into other cell compartments and how this situation influences the response of antioxidants in these cell compartments during drought stress. Thus, in order to understand the importance of antioxidants in the protection of plants against drought it is essential to study the subcellular distribution of ascorbate, glutathione, and ROS at the subcellular level. Such data can give valuable information about possible limitations of ascorbate and glutathione to protect plants in certain cell compartments (chloroplasts and peroxisomes) during drought stress which will remain undetected if measurements are performed with whole leaves.

This study was aimed to investigate the response of compartment specific ascorbate and glutathione contents in Arabidopsis plants during drought stress by computer supported transmission electron microscopy on a high level or resolution. The situation was monitored over a period of 10 days in order to investigate the dynamic subcellular protection of these key antioxidants against ROS produced during drought. Subcellular ascorbate and glutathione contents were compared between the wildtype plant *Arabidopsis thaliana* Col-0 and ascorbate and glutathione deficient mutants, *vtc2-1* (60% less ascorbate than the wildtype), [11] and *pad2-1* (80% less glutathione than the wildtype), [18], respectively, in order to investigate how plants with altered glutathione and ascorbate contents react to drought. Additional parameters such as H_2_O_2_ contents, pigment contents, photosynthesis, chlorophyll fluorescence, enzyme activity of glutathione reductase (GR), dehydroascorbate reductase (DHAR) and ascorbate peroxidase (APX) were monitored in order to correlate different defense and adaptation strategies of Arabidopsis plants to changes in the antioxidative protection during drought conditions.

## 2 Material and methods

### 2.1 Plant material

*A. thaliana* [L.] Heynh. ecotype Columbia (Col-0), the ascorbate and glutathione deficient mutants *vtc2-1* and *pad2-1*, respectively, were grown on “Naturahum” potting soil (Ostendorf Gärtnereierden GmbH., Vechta, Germany) in growth chambers with 8/16 h day/night photoperiod and a light intensity of 150 μmol m^−2^ s^−1^. Day and night temperatures were 22 °C and 18 °C, respectively, the relative humidity was set at 60% and the plants were cultivated at 100% relative soil water content. Six week old plants were subject to drought stress by withholding water for 10 days. At the time of harvesting all plants were 8 weeks old. Leaves were harvested from the fourth rosette and care was taken that they were about the same size and at similar developmental stage.

### 2.2 Determination of relative water contents, biomass and turgor pressure

Relative water contents (RWC) of leaves and soil was determined by subtracting the dry weight from the fresh weight of Arabidopsis leaves. Fresh weight was determined immediately after cutting the leaves from the plants whereas dry weight was determined after drying the leaves and the soil for 5 days at 90 °C. Biomass was determined by measuring the fresh weight of leaves and stems of control and drought stressed plants. Relative changes in turgor pressure were monitored using leaf patch clamp probes (LPCP) according to [19] in order to measure the relative turgor pressure of the leaves. Intact leaves of Arabidopsis Col-0, *pad2-1* and *vtc2-1* plants were positioned between two planar circular metal pads integrated into two magnets. Cell turgor pressure was measured by the pressure sensor chip integrated in the lower pad and determined as the output leaf patch pressure, *P*_p_, upon application of a constantly kept external clamp pressure. The minimum level of output leaf patch pressure is equivalent to the most turgescent state of the cells [19], [20]. Increase in output leaf patch pressure indicates a drop in turgor pressure.

### 2.3 Microscopical investigations of subcellular ascorbate, glutathione and H_2_O_2_

Sample preparation for immunogold labeling of ascorbate and glutathione, and visualization of H_2_O_2_ by cerium chloride (CeCl_3_) was performed as described previously [21], [22]. Briefly, sections of the youngest fully developed leaves were fixed in 2.5% paraformaldehyde and 0.5% glutaraldehyde for cytohistochemical investigations. Samples were then rinsed in buffer, dehydrated in increasing concentrations of acetone and gradually infiltrated with increasing concentrations of LR-White resin. Samples were finally polymerized in fresh pure LR-White at 50 °C for 48 h under anaerobic conditions. Sections for subcellular H_2_O_2_ visualization were incubated in 5 mM CeCl_3_ and then fixed in 2.5% glutaraldehyde. Samples were then rinsed in buffer, post-fixed in 1% osmium tetroxide, dehydrated in increasing concentrations of acetone and infiltrated with increasing concentrations of Agar 100 epoxy resin. Samples were finally polymerized in pure fresh resin at 60 °C for 48 h. Ultrathin sections (80 nm) were cut with a Reichert Ultracut S ultramicrotome (Leica Microsystems, Vienna, Austria).

Immunogold labeling of ascorbate and glutathione and evaluation of labeling through negative controls was done according to [11], [18]. Sections were blocked with 2% bovine serum albumine and then treated with the primary antibodies against ascorbate diluted 1:300 and glutathione diluted 1:50. After rinsing the sections in buffer, samples were incubated with secondary gold conjugated antibodies diluted 1:50 (for sections incubated with the glutathione antibody) and 1:100 (for sections incubated with the ascorbate antibody). After three washes in distilled water labeled grids were post stained with uranyl-acetate for 15 s and investigated with a Philips CM10 transmission electron microscope (TEM). Gold particles were counted using the software package Cell F in the different cell compartments. A minimum of 20 (peroxisomes and vacuoles) to 60 (other cell structures) sectioned cell structures of at least 15 different cells were analyzed. The specificity and accuracy of the immunogold localization methods has been demonstrated in detail in previous experiments [11], [18], [23], [24], [25], [26], [27], [28], [29].

### 2.4 Biochemical investigations

#### 2.4.1 Activity of APX, GR, DHAR

Enzyme activity was measured with a modified method according to [30]. Fresh leaf material from plant exposed for 14 days to different light treatments was ground in liquid nitrogen and incubated for 30 min on ice with 20 mg insoluble polyvinylpyrrolidone in extraction buffer (v/w) containing 100 mM NaH_2_PO_4_ (pH 7.5) and 1 mM EDTA. The homogenate was centrifuged at 4 °C until a clear supernatant was obtained. All reactions were carried out in a total volume of 500 μl in UV-permeable plastic cuvettes at 25 °C against reagent blank on a UV–VIS spectrophotometer (Hitachi U-3000). APX activity was measured as the decrease in absorbance at 290 nm due to ascorbate oxidation (*ɛ*_290_ = 2.8 mM^−1^ cm^−1^) in 100 mM NaH_2_PO_4_ (pH 7.5) buffer containing 1 mM EDTA, 0.2 mM H_2_O_2_, 0.5 mM Asc and enzyme extract. GR activity was assayed by following the oxidation of NADPH at 340 nm (*ɛ*_340_ = 6.22 mM^−1^ cm^−1^). Reaction contained 100 mM NaH_2_PO_4_ (pH 7.5), 1 mM EDTA buffer containing 0.1 mM NADPH, 0.5 mM GSSG and enzyme extract. DHAR was assayed in 100 mM NaH_2_PO_4_ (pH 7.0), 1 mM EDTA buffer containing 0.2 mM dehydroascorbate, 2.5 mM GSH and plant extract by the increase in absorbance at 265 nm (*ɛ*_265_ = 14 mM^−1^ cm^−1^).

#### 2.4.2 Determination of pigments

Chloroplast pigments (neoxanthin, violaxanthin, lutein, zeaxanthin, chlorophyll a, chlorophyll b and β-carotene) were separated and determined in one step using HPLC (high-performance liquid chromatography) gradient method (modified according to [31]). Leaves were frozen and ground in liquid nitrogen and pulverized plant-material (60 mg) was added to 60 mg calcium carbonate and extracted with DMSO/ethanol (Dimethylsulfoxide, 2:1, v:v) three times on ice in the dark. Samples were then centrifuged for 30 min at 4 °C at 14,000 rpm. Separation and determination of the pigments was done on a gradient HPLC (HP Chemstation, 4 °C cooled autosampler, used column 25 × 4.6 mm Grom Spherisorb ODS2 5 μm, photometric detection by HP diode array detector 1040 M at 440 nm). Solvent A: acetonitrile/aqua bidest./methanol (100/10/5, v/v/v); Solvent B: acetone/ethyl acetate (2/1, v/v); Gradient: 10–80% solvent B in 18 min. Flow rate: 1 ml min^−1^.

### 2.5 Photosynthetic activity

#### 2.5.1 Chlorophyll fluorescence

Chlorophyll fluorescence was measured using a PAM-2000 (Walz, Effeltrich, Germany), a pulse amplitude modulation fluorometer. After dark adaptation of at least 20 min using leaf clips Fv/Fm was determined and after illumination with actinic light for 3 min Fm′ was determined. Nonphotochemical quenching (NPQ) was calculated (NPQ = (Fm−Fm′)/Fm′) as described previously [32].

#### 2.5.2 Gas exchange

Photosynthetic parameters (net photosynthesis, transpiration and stomatal conductance) were measured by a LI-6400 portable photosynthesis system (LI-COR, Lincoln, USA) using the extended reach 1 cm chamber, a CO_2_ reference concentration of 400 μmol ml^−1^ at a flow rate of 300 μmol s^−1^. Water use efficiency (WUE) was calculated as net photosynthesis/transpiration rate.

## 3 Results

### 3.1 Visible symptoms

**Fig. 1:**
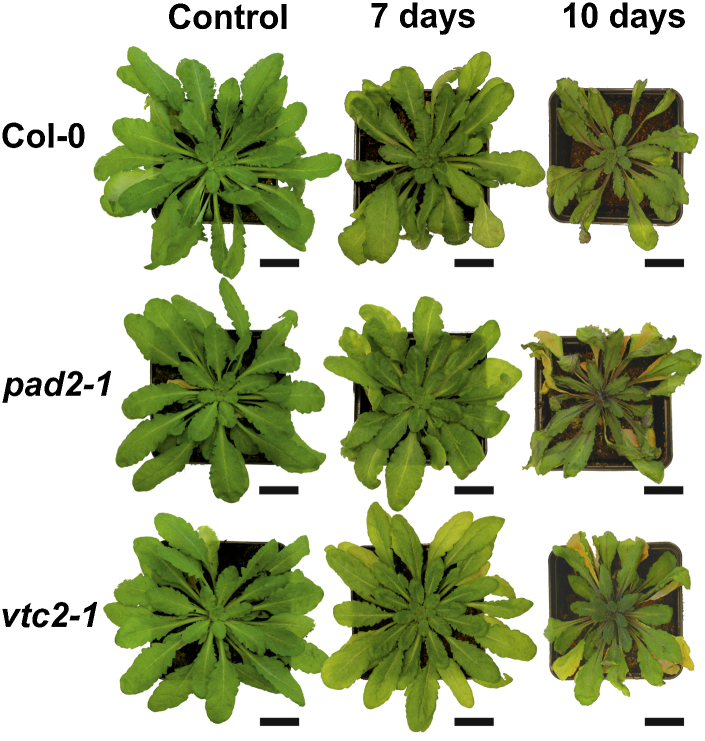
Col-0, *pad2-1* and *vtc2-1* during drought stress. Representative images of plants from *Arabidopsis thaliana* Col-0 (first row), and the mutants *pad2-1* (second row) and *vtc2-1* (third row) grown under drought stress conditions for 10 days. First signs of drought induced changes could be found 7 days after the stop of irrigation when older leaves showed light chlorosis. Ten days after the stop of irrigation older leaves showed strong wilting, chlorosis and necrosis whereas younger leaves did not show any visible signs of drought stress. Bar = 1 cm.

Drought stress severely affected the growth and the phenotype of the plants. First visible drought stress induced symptoms (yellowing of leaves) could be observed when plants were exposed to drought for 7 days (Fig. 1). When plants were exposed to drought for 10 days some leaves showed advanced yellowing and the majority of the older leaves showed severe wilting. In addition necrosis could be observed on some leaves of the mutants (Fig. 1). Such symptoms could not be observed in leaves of the youngest developed rosette. Wildtype plants showed less severe symptoms than the mutants and did not develop necrosis (Fig. 1).

### 3.2 Relative water contents, biomass and turgor pressure

**Fig. 2:**
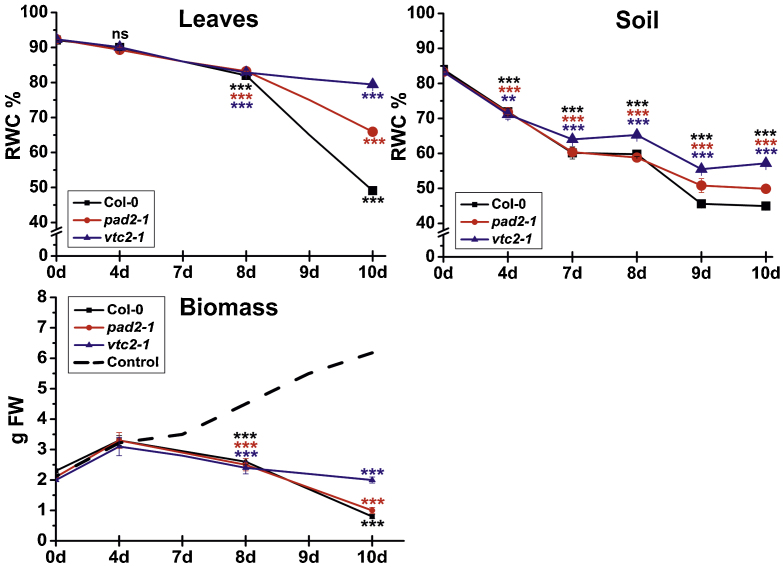
Relative water content of soil and leaves and plant biomass during drought stress. Graphs show relative water contents (RWC) in percent in leaves and soil of wildtype plants, and biomass in (fresh weight in g) of *pad2-1* and *vtc2-1* mutants during drought stress over a period of 10 days. Data are means with standard errors. Significant differences were calculated between control and drought conditions with a *t*-test; ^***^, ^***^, indicates significance at the 0.001 levels of confidence.

RWC strongly decreased in leaves of Arabidopsis Col-0 plants and the mutants (Fig. 2). At the beginning of the experiment a RWC of about 92% was measured in these plants. It dropped to about 82%, 8 days after the stop of irrigation and to 49%, 66% and 79% in Col-0, *pad2-1* and *vtc2-1* at the end of the experiment (Fig. 2). These data correlated well with a strong decrease in RWC of the soil. At the beginning of the experiment soil water content was about 85% and dropped to about 70%, 4 days after the stop of irrigation. Soil water contents reached about 45%, 50% and 55% in the soil of Col-0, *pad2-1* and *vtc2-1* plants at the end of the experiment (Fig. 2). Determination of the biomass of plants revealed no significant difference between well watered wildtype plants and the mutants (Fig. 2). While similar biomass values were found for the first 8 days of drought (2.5 g) *vtc2-1* mutants showed higher fresh weight (2 g) at the end of the experiment than Col-0 and *pad2-1* (0.8 and 1 g, respectively).

**Fig. 3:**
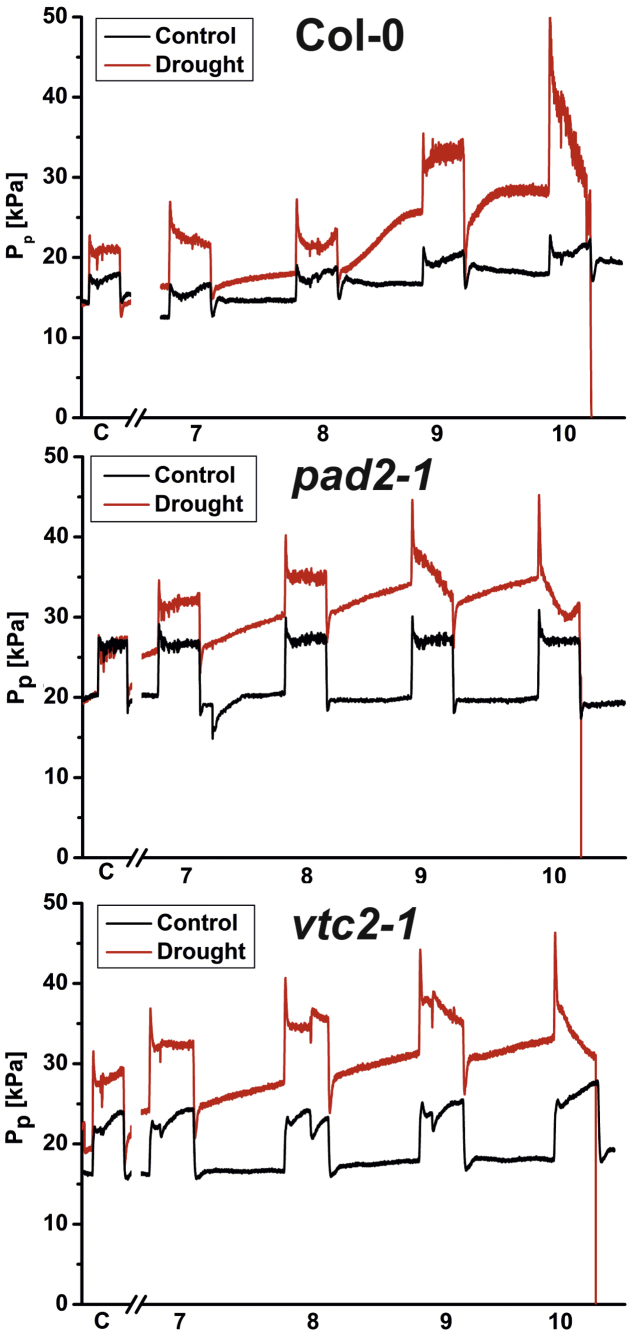
Diurnal *P*_P_ changes in leaves during drought stress. Graphs show changes in output clamp pressure (*P*_P_) on leaves of *A. thaliana* Col-0 plants and the Arabidopsis mutants *pad2-1* and *vtc2-1* at different time points during drought stress. Leaves under control conditions (black line) show a similar diurnal change in *P*_P_ with higher *P*_P_ at day time (lower turgor pressure) and lower *P*_P_ at night time (higher turgor pressure) throughout the experiment. Leaves of drought stressed plants (red lines) showed a continuous raise starting at day 7 in *P*_P_ when compared to the well watered situation at the beginning of the experiment (C). Ten days after the stop of irrigation *P*_P_ was highest right after the beginning of day light with a strong drop throughout the day until no measurements were available anymore due to plant death. (For interpretation of the references to color in this figure legend, the reader is referred to the web version of this article.)

Relative changes in turgor pressure with LCPC revealed similar diurnal changes in well watered Arabidopsis Col-0 plants and the *vtc2-1* and *pad2-1* mutants over the whole course of the experiment (Fig. 3). Output leaf patch pressure (*P*_p_) was lowest in darkness and highest during illumination indicating full turgescence of the cells at night time and a loss of turgor pressure during illumination at day time. Drought stressed plants showed similar diurnal changes in leaf patch pressure at the beginning of the experiment. Leaf patch pressure showed a continuous increase with drought conditions indicating the loss of turgor pressure (Fig. 3). This increase could be best observed at night time around 7 days after the stop of irrigation indicating that the plants were not able to recover from cell turgor loss of transpiring leaves during the night. Ten days after the stop of irrigation leaf patch pressure reached highest levels in all plants before it started to drop to zero levels right after the onset of illumination indicating the death of the plant (Fig. 3).

### 3.3 Microscopical investigations

#### 3.3.1 Ascorbate

In most cell compartments the subcellular distribution of ascorbate in control plants was similar to what has been reported previously [11]. Whereas wildtype plants and *pad2-1* mutants showed similar ascorbate levels in all cell compartments the *vtc2-1* mutant contained between 35% and 81% less ascorbate (chloroplasts and mitochondria, respectively) than the wildtype (Supplementary Table 1 and Supplementary Figs. A1–A3). These results are similar to what has been described in recent studies which revealed that the *vtc2-1* mutant contained between 50 and 60% less ascorbate than wildtype plants [11].

**Fig. 4:**
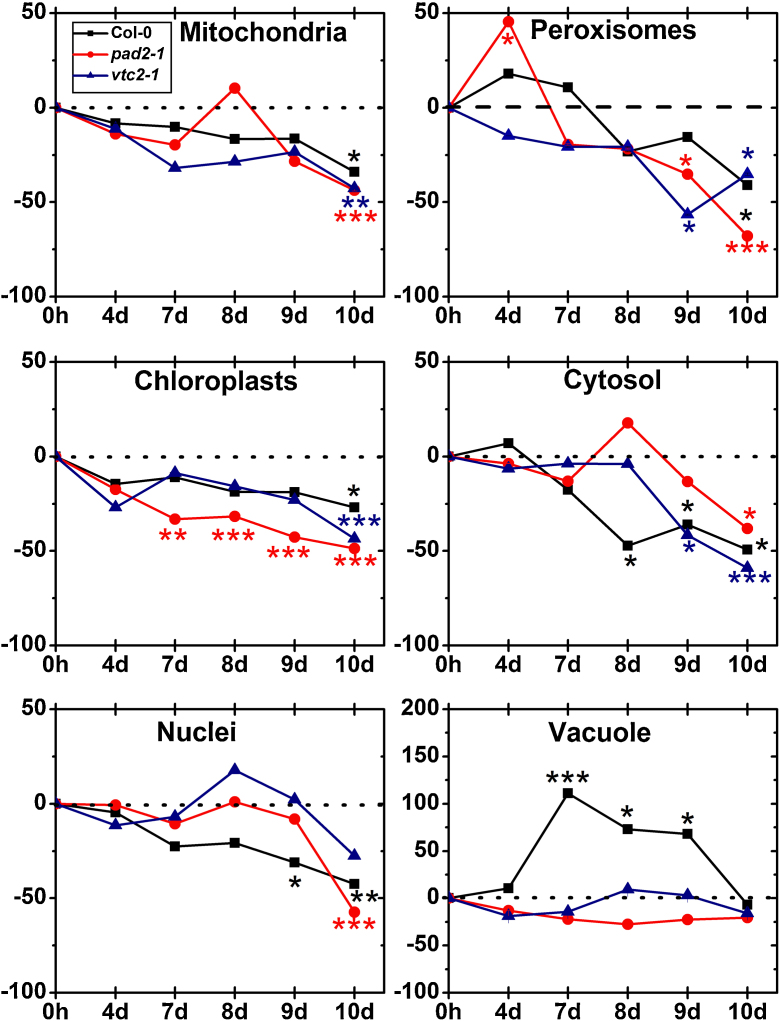
Compartment specific ascorbate labeling during drought stress. Graphs show changes (in percent) in the amounts of gold particles bound to ascorbate per μm^2^ in mesophyll cells of *A. thaliana* Col-0 plants (black squares) and the Arabidopsis mutants *pad2-1* (red circles) and *vtc2-1* (blue triangles) during drought over a period of 10 days when compared to the control. *n* > 20 for peroxisomes and vacuoles and *n* > 60 for other cell structures. Data are means with standard errors and are based on data shown in Supplementary Table 1. Significant differences were calculated between control and drought conditions with the Mann Whitney *U*-test; ^*^, ^**^ and ^***^, respectively, indicate significance at the 0.05, 0.01 and 0.001 levels of confidence. (For interpretation of the references to color in this figure legend, the reader is referred to the web version of this article.)

Ascorbate contents in wildtype plants showed a strong increase in vacuoles when exposed to drought for 7 (111%), 8 (73%) and 9 days (68%) (Fig. 4). In the other cell compartments of Col-0 significant changes were not observed until 8 days after the exposure to drought when the cytosol showed a strong decrease of 47% and in ascorbate contents. Significant decreased levels of ascorbate were found in mitochondria (43%), chloroplasts (44%), peroxisomes (35%) and the cytosol (65%) when wildtype plants were exposed to drought for 10 days (Fig. 4; Supplementary Fig. A1). *pad2-1* mutants showed a strong decrease in ascorbate contents in chloroplast (between 33 and 44%) when exposed to drought between 7 and 10 days. Peroxisomes of the *pad2-1* mutant contained about 68% less ascorbate specific labeling when exposed to drought for 10 days (Fig. 4). Other cell compartments (except vacuoles which showed unchanged levels throughout the drought stress experiment) contained less ascorbate contents only when *pad2-1* mutants were exposed to drought for 10 days (Fig. 4; Supplementary Fig. A2).

Drought did not affect ascorbate contents in nuclei and vacuoles of *vtc2-1* mutants (Fig. 4). Changes in compartment specific ascorbate contents could be observed in peroxisomes (up to 57%) and the cytosol (up to 65%) when exposed to drought for 9 and 10 days (Fig. 4). In mitochondria and chloroplasts of the *vtc2-1* mutant ascorbate contents significantly decreased by about 43% when exposed to drought for 10 days (Fig. 4; Supplementary Fig. A3).

#### 3.3.2 Glutathione

Compartment specific glutathione contents in control plants was similar in most cell compartments as described recently [28]. Subcellular glutathione contents were similar in wildtype plants and the *vtc2-1* mutants whereas the *pad2-1* mutant contained between 80 and 93% less glutathione labeling (chloroplasts and nuclei, respectively) than the wildtype (Supplementary Table 2; Supplementary Figs. A3–A5). Only mitochondria in *pad2-1* mutants contained similar glutathione levels when compared to the wildtype. These results are similar to what has been shown in recent studies where mitochondria of the *pad2-1* mutant contained wildtype glutathione levels whereas all other cell compartments contained up to 90% less glutathione than the wildtype [18]. Additionally, it has been shown in previous studies that *vtc2-1* mutants contained similar glutathione levels when compared to the wildtype [33].

**Fig. 5:**
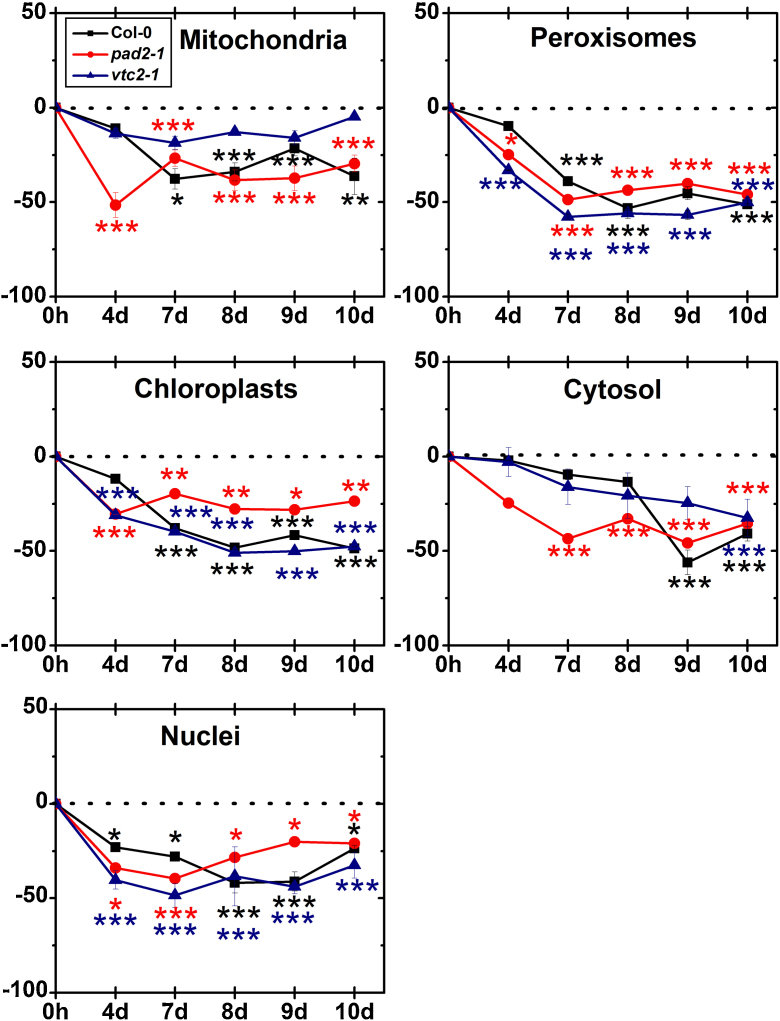
Compartment specific glutathione labeling during drought stress. Graphs show changes (in percent) in the amounts of gold particles bound to glutathione per μm^2^ in mesophyll cells of *A. thaliana* Col-0 plants (black squares) and the Arabidopsis mutants *pad2-1* (red circles) and *vtc2-1* (blue triangles) during drought over a period of 10 days when compared to the control. *n* > 20 for peroxisomes and vacuoles and *n* > 60 for other cell structures. Data are means with standard errors and are based on data shown in Supplementary Table 2. Significant differences were calculated between control and drought conditions with the Mann Whitney *U*-test; ^*^, ^**^ and ^***^, respectively, indicate significance at the 0.05, 0.01 and 0.001 levels of confidence. (For interpretation of the references to color in this figure legend, the reader is referred to the web version of this article.)

The exposure of wildtype plants to drought for 4 days induced significant decreased levels of glutathione (23%) only in nuclei whereas glutathione contents in all other cell compartments remained at control levels (Fig. 5). All cell compartments contained less glutathione (up to 38% in mitochondria, 51% in chloroplasts, 41% in nuclei, 53% in peroxisomes and 56% in the cytosol) when exposed to drought for 7 days or longer (Fig. 5; Supplementary Fig. A4).

When the glutathione deficient *pad2-1* mutants were exposed to drought stress a first significant decrease of glutathione contents could be observed in most cell compartments (51% in mitochondria, 31% in chloroplasts, 34% in nuclei and 28% in the cytosol) 4 days after withholding water (Fig. 5). Glutathione contents remained at similar low levels in all cell compartments of the *pad2-1* mutants exposed to drought between 7 and 10 days (Fig. 5; Supplementary Fig. A5).

A first significant decrease in glutathione contents of the *vtc2-1* mutants was observed in chloroplasts (31%), nuclei (40%) and the cytosol (33%) when exposed to drought for 4 days (Fig. 5). Glutathione contents decreased further in these cell compartments to 48% in chloroplast, 50% in peroxisomes, 33% in nuclei and the cytosol when *vtc2-1* mutants were exposed to drought for 10 days. Glutathione contents remained at control levels in mitochondria of the *vtc2-1* mutant during the whole period of investigation (Fig. 5; Supplementary Fig. A6).

#### 3.3.3 H_2_O_2_ accumulation

**Fig. 6:**
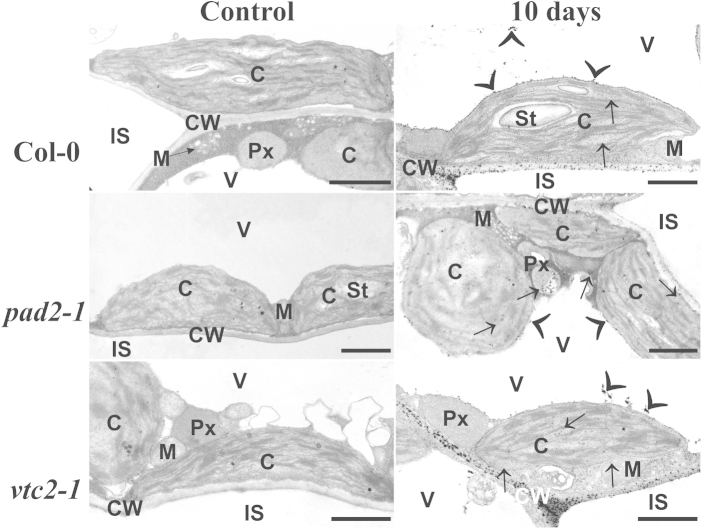
Subcellular visualization of H_2_O_2_ in leaves during drought stress. TEM-micrographs show the subcellular distribution of H_2_O_2_ visualized by CeCl_3_-staining in leaf cells of *A. thaliana* [L.] Heynh. ecotype Columbia (Col-0) and the mutants *pad2-1* and *vtc2-1* exposed to drought stress for 10 days. Strong CeCl_3_-staining along the tonoplast, inside vacuoles (arrowheads), the cytoplasm (arrows), and cell walls (CW) was observed when plants were exposed to drought stress for 10 days. Controls showed no or only very little staining. C = chloroplasts with or without starch (St), M = mitochondria, Px = peroxisomes, IS = intercellular spaces and V = vacuoles. Bars = 1 μm.

H_2_O_2_ accumulation visualized by CeCl_3_-staining revealed that 10 days after the stop of irrigation it occurred in high quantities in cell walls of wildtype plants and both mutants. H_2_O_2_ was also detected along the tonoplast and inside vacuoles (Fig. 6). CeCl_3_ precipitation was also found in chloroplasts, mitochondria, peroxisomes and the cytosol indicating that H_2_O_2_ also accumulates in the cytoplasm during drought stress (Fig. 6).

### 3.4 Biochemical investigations

#### 3.4.1 Enzyme activity

**Fig. 7:**
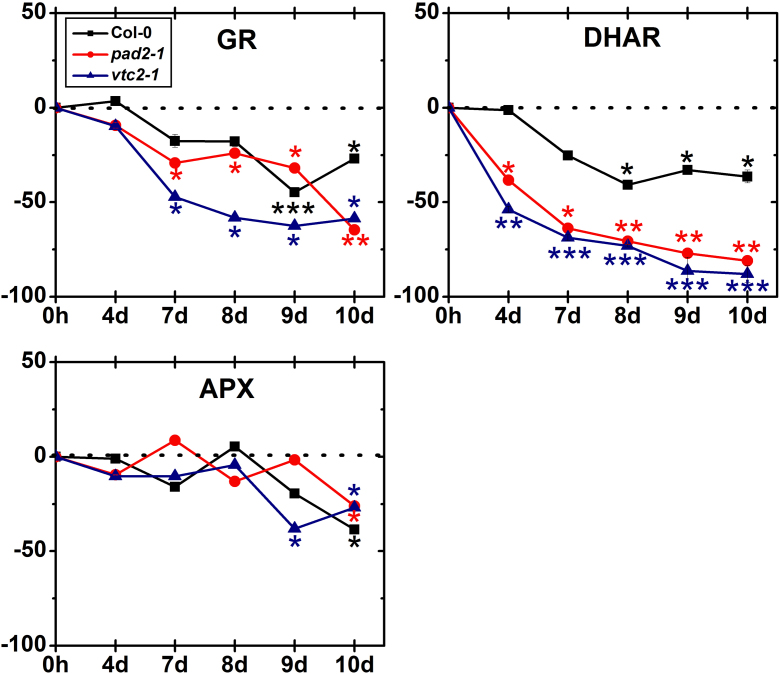
Activity of antioxidative enzymes. Graphs show changes (in percent) in activities of GR, DHAR and APX in leaves of wild type (Col-0) plants, the *pad2-1* and the *vtc2-1* during drought stress over a time period of 10 days when compared to the control. Data are means with standard errors and are based on data shown in Supplementary Table 3. *n* = 9. Different lowercase letters indicate Significant differences were calculated between control and drought conditions with the Mann Whitney *U*-test; ^*^, ^**^ and ^***^, respectively, indicate significance at the 0.05, 0.01 and 0.001 levels of confidence.

In control plants the activity of GR was similar in Col-0 and *vtc2-1*, but lower (21%) in the *pad2-1* mutant (Supplemental Table 3). Whereas GR activity in wildtype plants significantly decreased (55% and 27%) when exposed to drought for 9 and 10 days, respectively, *pad2-1* and *vtc2-1* mutants showed 29% and 47% less GR activity already 4 days after the exposure to drought. Activity further decreased in these mutants of about 64% in *pad2-1* and 59% in *vtc2-1* when exposed to drought for 10 days (Fig. 7). At the end of the experiment wildtype plants showed the highest activity of DHAR when compared to the mutants.

Under control conditions *vtc2-1* mutant showed higher APX activity than wildtype plants (17%) and *pad2-1* mutants (29%) (Supplementary Table 3). APX activity was significantly decreased (38%) in *vtc2-1* mutants exposed to drought for 9 days whereas unchanged activity was found in wildtype plants and the *pad2-1* mutant (Fig. 7). A significant decrease of ascorbate peroxidase activity was then found in all plants when exposed to drought for 10 days (39% in wildtype plants, 27% in *pad2-1* and *vtc2-1* mutants).

In control plants DHAR activity was highest in wildtype plants and lower in *pad2-1* (29%) and *vtc2-*1 mutants (71%) (Supplementary Table 3). DHAR activity was significantly decreased of up to 41% when wildtype plants were exposed to drought between 8 and 10 days (Fig. 7). A strong decrease of DHAR activity was first detected in *pad2-1* and *vtc2-1* mutants when exposed to drought for 4 days (38% and 54%, respectively). Activity decreased to 81% in *pad2-1* and 88% in *vtc2-1* when exposed to drought for 10 days (Fig. 7). At the end of the experiment wildtype plants showed the highest activity of DHAR when compared to the mutants.

#### 3.4.2 Pigment contents

**Fig. 8:**
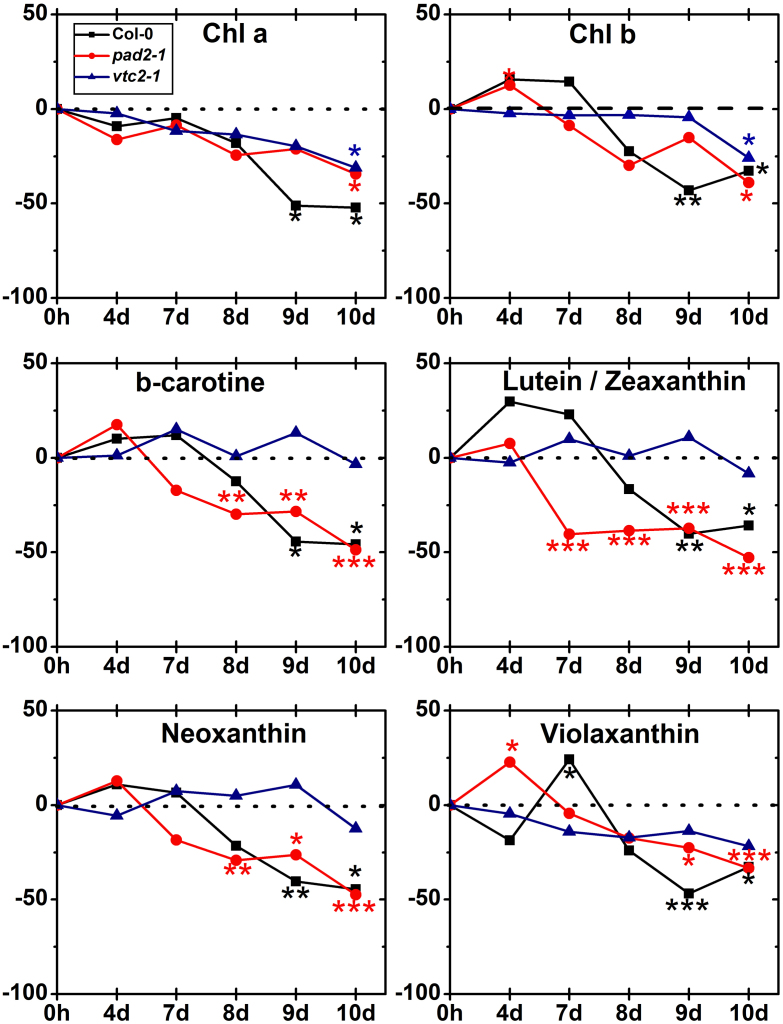
Contents of chlorophyll a and b during drought stress. Graphs show changes in the contents of chlorophyll a and b in percent in leaves of wildtype plants, *pad2-1* and *vtc2-1* mutants during drought stress over a time period of 10 d. Data are means with standard errors and are based on data shown in Supplementary Table 4. Significant differences were calculated between control and drought conditions with a *t*-test; ^*^, ^**^ and ^***^, respectively, indicate significance at the 0.05, 0.01 and 0.001 levels of confidence. ns = not significant different. *n* = 6.

Under control conditions chlorophyll a and b contents were similar in wildtype plants and both mutants (Supplementary Table 4). Contents of chlorophyll a and b was strongly decreased 9 and 10 days after the stop of irrigation in wildtype plants (up to 52% for chl a and 43% for chl b), and 10 days after the stop of irrigation in both mutants (up to 31% for chl a and 39% for chl b). Contents of β-carotene, lutein/zeaxanthin, and neoxanthin was higher in the *pad2-1* mutant and always lower in wildtype plants. Violaxanthin was higher in *vtc2-1* mutants and lower in *pad2-1* mutants (39%) (Supplementary Table 4). Whereas β-carotene, lutein/zeaxanthin, neoxanthin, and violaxanthin were strongly decreased in wildtype plants and the *pad2-1* mutant during drought conditions (starting 7 days after the stop of irrigation in *pad2-1*) unchanged levels were found in *vtc2-1* mutants when compared to the control (Fig. 8). In wildtype plants a strong decrease in β-carotene (up to 46%), lutein/zeaxanthin (up to 37%), neoxanthin (up to 45%) and violaxanthin (up to 47%) could be observed starting 9 days after the stop of irrigation (Fig. 8). *pad2-1* mutants showed up to 49% less β-carotene and neoxanthin starting 8 days after the stop of irrigation and up to 53% and 33% less lutein/zeaxanthin and violaxanthin starting at 7 and 9 days after the stop of irrigation, respectively (Fig. 8).

#### 3.4.3 Photosynthetic efficiency

**Fig. 9:**
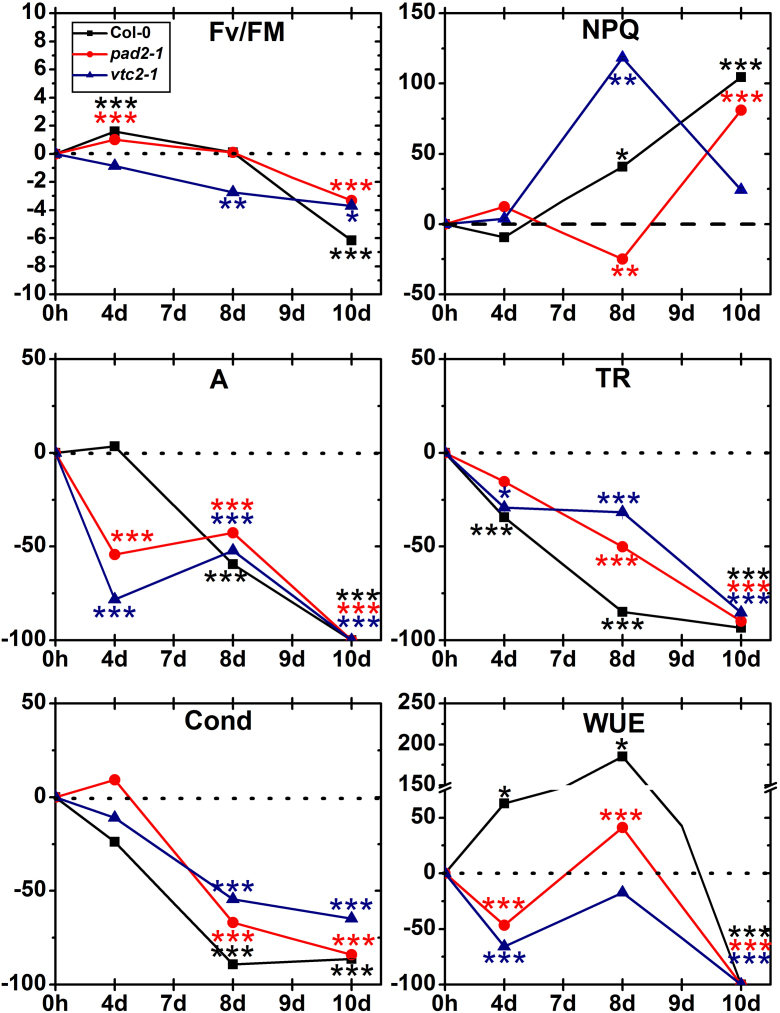
Photosynthetic efficiency. Graphs show changes (in percent) in chlorophyll fluorescence (Fv/Fm, NPQ), net photosynthesis (A), stomatal conductance (Cond), transpiration rate (TR), and water use efficiency (WUE) in leaves of wild type (Col-0) plants, the *pad2-1* and the *vtc2-1* during drought stress over a time period of 10 days when compared to the control. Data are means with standard errors and are based on data shown in Supplementary Table 5. *n* = 10. Different lowercase letters indicate Significant differences were calculated between control and drought conditions with the Mann Whitney *U*-test; ^*^, ^**^ and ^***^, respectively, indicate significance at the 0.05, 0.01 and 0.001 levels of confidence.

Well watered control plants showed similar Fm/Fv values (Supplementary Table 5). While Col-0 plants and *pad2-1* mutants showed significant increased Fm/Fv values (up to 1.5%) 4 days after the stop of irrigation unchanged values were found at the *vtc2-1* mutant at this stage (Fig. 9). A strong decrease could be observed in the *vtc2-1* mutants 8 and 10 days (2.7% and 3.7%, respectively) after the stop or irrigation when compared to control plants. Col-0 plants and *pad2-1* plants showed significant lower Fm/Fv values (3.3% and 6.2%, respectively) 10 days after the stop of irrigation (Fig. 9). In control plants NPQ values of the *vtc2-1* mutant were much lower when compared to Col-0 (49%) and *pad2-1* mutants (42%) (Supplementary Table 5). A significant increase of NPQ values in Col-0 plants and *vtc2-*1 mutants (41% and 118%, respectively) could be observed 8 days after the stop of irrigation whereas *pad2-1* mutants showed a decreased value (25%) when compared to well watered control plants. All plants showed elevated NPQ levels at the end of the drought stress experiment (Fig. 9). The highest increase was found in Col-0 (105%), followed by *pad2-1* (81%) and *vtc2-1* (24%).

Net photosynthesis in well watered control plants was similar in wildtype plants and *pad2-1* mutants. In comparison to wildtype plants *vtc2-1* mutants showed about 48% less net photosynthesis under control conditions (Supplementary Table 5). Both mutants showed decreased (54% in *pad2-1* and 78% in *vtc2-1*) net photosynthesis starting 4 days after the stop of irrigation while a first decrease in net photosynthesis in wildtype plants could be observed 8 days after the stop of irrigation (Fig. 9). Photosynthetic activity could not be measured at the end of the experiment. In well watered control plants transpiration rate was similar in all plants (Supplementary Table 5). Decreased transpiration rate could be observed in all plants throughout the experiment starting at 4 days after the stop of irrigation (15% in *pad2-1*, 29% in *vtc2-1* and 35% in Col-0) reaching the lowest values at the end of the experiment (decrease between 85 and 93%) (Fig. 9). Stomatal conductance was significantly decreased in all plants starting 8 days after the onset of drought (up to 89% in Col-0, 67% in *pad2-1* and 55% in *vtc2-1*) when compared to well watered control plants (Fig. 9). Under control conditions all plants showed a similar stomatal conductance (Supplementary Table 5). In well watered control plants WUE was highest in *pad2-1* mutants (22% than in Col-0) and lowest in *vtc2-1* mutants (42% lower than in Col-0) (Supplementary Table 5). During the first 8 days of drought stress WUE was strongly increased in wildtype plants (up to 185% 8 days after the stop of irrigation) and decreased in *vtc2-1* mutants (up to 66% 4 days after the stop of irrigation). WUE of the *pad2-1* mutant decreased at the beginning (47% 4 days after the stop of irrigation) and increased 8 days after the stop of irrigation (41%) when compared to well watered control plants (Fig. 9). A positive net photosynthesis could not be measured after 10 days of drought stress. Therefore WUE decreased to zero levels in all plants.

## 4 Discussion

The results obtained in this study revealed several important aspects on the compartment specific protection of antioxidants during drought. Based on the data of RWC of the leaves, biomass and LCPC first signs of drought stress in the leaves could be observed around 7 days after the stop of irrigation when RWC, biomass and turgor pressure in the leaves started to drop when compared to the control. Nevertheless, a drop in glutathione contents could already be observed 4 days after the stop of irrigation in peroxisomes, chloroplasts and nuclei in both mutants but not in wildtype plants demonstrating that the mutant plants reacted more sensitive to drought stress than Col-0. The reasons therefore are not clear yet but it could be related to a weaker antioxidative capacity in these mutants due to lower glutathione (*pad2-1* contains about 80% less glutathione than the wildtype) and ascorbate (*vtc2-1* contains about 50% less ascorbate than the wildtype) contents [18], [22], [27]. At this time point the RWC of the soil decreased from 85% at the beginning of the experiment to about 75% in drought stressed plants. These results indicate that roots sensed the decrease in water contents of the soil and signaled it to the leaves at a stage where drought stress was not yet measurable in leaves as RWC and LCPC measurements did not differ from the control conditions. Ascorbate contents remained unchanged in the mutants at this stage indicating that it does not play important roles in signaling drought stress from roots to leaves. The drop in glutathione contents in leaves 4 days after the stop of irrigation is especially interesting as the interplay between ROS and antioxidants in chloroplasts (and peroxisomes) are important for signals that are sent to the nucleus during stress conditions. Such retrograde signals can either trigger programmed cell death or are involved in the adaptation to environmental changes (reviewed in [34]). In the case of drought such responses could include the repression of genes involved in photosynthesis [34], [35] and the control of stomata closure as it was observed that the depletion of glutathione is involved in stomata closure [36], [37]. The latter could not be confirmed 4 days after the stop of irrigation as a similar decrease in stomatal conductivity and transpiration rate was observed in all plants independent of the glutathione status. Net photosynthesis and WUE was lower in the mutants when compared to wildtype plants at this time point. These results indicate that the mutants showed higher sensitivity to drought stress in terms of lower photosynthetic activity at the beginning of the experiment which might be related to lower antioxidative capacity in ascorbate and glutathione deficient mutants especially in chloroplasts and peroxisomes when compared to the wildtype plants. At later time points of drought stress stomata closure and down-regulation of photosynthesis could be observed which are both common responses of plants to drought [34], [35], [36], [37].

In the long term a general decrease of glutathione could be observed in all plants in chloroplasts, peroxisomes and nuclei starting 7 days after the stop of irrigation until the end of the experiment. At later time points (9 and 10 days after the stop of irrigation) ascorbate contents also decreased in these cell compartments. At all of these time point RWC of both soil and leaves showed a strong decrease. Additionally, LCPC measurements showed a decrease in turgor pressure in all plants indicating that drought stress occurred in the leaves. Under these conditions low CO_2_ in chloroplasts induced by stomata closure during drought induces malfunctions of the Calvin cycle and will favor a process called photorespiration which leads to the production of H_2_O_2_ in peroxisomes [1], [2], [3], [4]. Thus, the drop of ascorbate and glutathione in these cell compartments indicates that large amounts of ascorbate and glutathione were needed and used in order to counteract ROS-accumulation in chloroplasts and peroxisomes. Low ascorbate and glutathione contents at later stages of drought correlated with a suppressed activity of enzymes involved in ascorbate-glutathione cycle such as GR, APX and DHAR. Low activities of these enzymes will shift the ascorbate and glutathione pool more towards their oxidized forms which will decrease the ability of the plant to detoxify ROS. This, decreased antioxidative capacity will then lead to an accumulation of ROS in the tissue which has been observed in Arabidopsis during drought stress [13]. In this respect it is interesting that wildtype plants showed much higher activity of GR and DHAR than the mutants at the end of the experiment which correlated with highest NPQ levels of all plants and weaker symptom development and the absence of necrosis in wildtype plants. These results demonstrate that wildtype plants were better adapted to drought stress than the mutants as they had a higher capacity to dissipate excess light energy under severe drought conditions.

In this study we could visualize that H_2_O_2_ accumulated specifically in chloroplasts, cell walls and the cytosol and correlated with a general decrease of ascorbate and glutathione contents. These data demonstrates that in drought stressed leaves low ascorbate and glutathione contents in these cell compartments were not able to keep ROS accumulation under control. An accumulation of ROS in plants will lead to degradation of nucleic acids, lipids, pigments, membranes, proteins, RNA and DNA, causing mutation and eventually cell death [6], [7], [8]. Such effects have also been found during this experiment as drought stressed plants at the later stages developed chlorosis (7 days after stop of irrigation) and necrosis (10 days after stop of irrigation) on the leaves and as pigments contents strongly decreased at the later stages of drought stress. A decrease in pigments especially chlorophyll has also been observed in other studies during drought in different plant species and is considered to be a stress marker for drought stress [13], [38], [39]. Whereas glutathione could not be detected in vacuoles of the plants exposed to drought, a strong increase in ascorbate contents (up to 111%) could be observed in vacuoles of wildtype plants during drought stress. This increase in wildtype plants was accompanied with a strong accumulation of H_2_O_2_ in vacuoles but also in cell walls and the cytoplasm. These results are similar to what has been described during excess light conditions and pathogen infection where a strong increase of ascorbate in vacuoles (up to nearly 400% and 111%, respectively) correlated with the accumulation of H_2_O_2_ in this cell compartment [21], [40]. Thus, it seems that the leakage of H_2_O_2_ from chloroplast and peroxisomes which are considered to be the main production center for ROS during drought stress [1], [2], [3], [4], [5] through the cytosol into the vacuoles seems to be a common mechanism during extreme stress conditions and indicates that vacuoles act as a sink for H_2_O_2_ during environmental stress situations. In vacuoles ascorbate helps to reduce phenoxyl radicals (created by oxidation of phenols by H_2_O_2_) and is oxidized to mono- and dehydroascorbic acid which is then transported into the cytosol for reduction to ascorbic acid [21], [41].

In this context it is interesting that *vtc2-1* mutants in opposite to the wildtype and *pad2-1* mutant did not show a decrease in β-carotene, lutein/zeaxanthin, neoxanthin and violaxanthin contents during drought stress. Xanthophylls and carotenoids protect plants against increased ROS production through various mechanisms [32], [40]. Especially during stress situations such as drought they become essential as they participate in the quenching of excess energy dissipation caused by overstraining the pathways of photosynthesis under such conditions [42]. Surprisingly, higher pigment contents did not lead to higher stress tolerance in *vtc2-1* mutants when compared to the wildtype and the *pad2-1* mutant. Investigations of photosynthetic efficiency revealed that *vtc2-1* mutants reacted even more sensitive to drought stress as a significant decrease of Fv/Fm values could be observed at the early stages of drought stress when Col-0 and *pad2-1* mutants showed unchanged or even slight increased values when compared to controls. Additionally, throughout the experiment *vtc2-1* mutants showed much lower NPQ values (up to 70% at the end of the experiment) than the wildtype, despite a strong increase 8 days after the stop of irrigation. Thus, it can be concluded that despite higher pigment contents *vtc2-1* mutant did not show higher photosynthetic activity or increased NPQ than Col-0 and *pad2-1* during drought stress. This observation could be explained by a lack of ascorbate in chloroplasts of the *vtc2-1* mutants when compared to the other plants as ascorbate is needed as a reducing agent during the conversation of violaxanthin to zeaxanthin in the xanthophyll cycle during NPQ [32].

In this study glutathione and ascorbate contents were found to be decreased during drought stress. These results are partly similar to other studies but also differ from results obtained with Arabidopsis under drought stress [13]. Differences can be explained by the fact that in this study glutathione and ascorbate contents were monitored over a time period of 10 days of withholding water. In other studies where a strong increase in total glutathione and a small increase in total ascorbate contents were found in wildtype plants, drought was induced rapidly within 3 days [13]. Interestingly *vtc2-1*showed a decrease in total ascorbate contents similar to what was found in this study. In another study where mild water stress was induced in *vtc2-1* mutants over a time period of 3 weeks unchanged total glutathione contents and a decrease of total ascorbate contents was observed at the end of the drought stress experiment [14]. Similar results were obtained for these plants when drought stressed was applied over a time period of 3 weeks as glutathione contents remained unchanged in wildtype plants and the *vtc2-1* mutants under these conditions [15]. Nevertheless, ascorbate contents strongly increased during drought stress in these plants. From these studies it becomes obvious that plants react differently to drought depending on the severity and length of drought stress and most probably also on soil conditions.

The results of this study demonstrated that wildtype plants were better adapted to drought conditions than the mutants as they showed less visible symptoms, an absence of necrosis and highest NPQ levels at the end of the experiment. These effects were most probably due to lower antioxidative capacity of the mutants which in addition to lower ascorbate and glutathione contents showed less activity of antioxidative enzymes such as GR and DHAR than the wildtype throughout the experiment. Glutathione acted as a signaling agent of drought stress in leaves of the mutants at early time points where drought stress was not yet measurable in leaves but could be detected by a slight drop of RWC in the soil. Such roles could not be attributed to ascorbate contents which remained unchanged in most cell compartments until very late stages of drought stress. At later time points the strong decrease of ascorbate and glutathione contents in chloroplasts and peroxisomes and the accumulation of H_2_O_2_ in the cells indicated that these antioxidants are depleted due to ROS accumulation in these cell compartments induced by malfunctions of the Calvin cycle and photorespiration. The strong increase of ascorbate contents in vacuoles in wildtype plants could be correlated with the accumulation of H_2_O_2_ in this cell compartment indicating that ascorbate plays an important role for the detoxification of H_2_O_2_ leaking from chloroplasts and peroxisomes into vacuoles during drought stress.

## Figures and Tables

**Figure:**
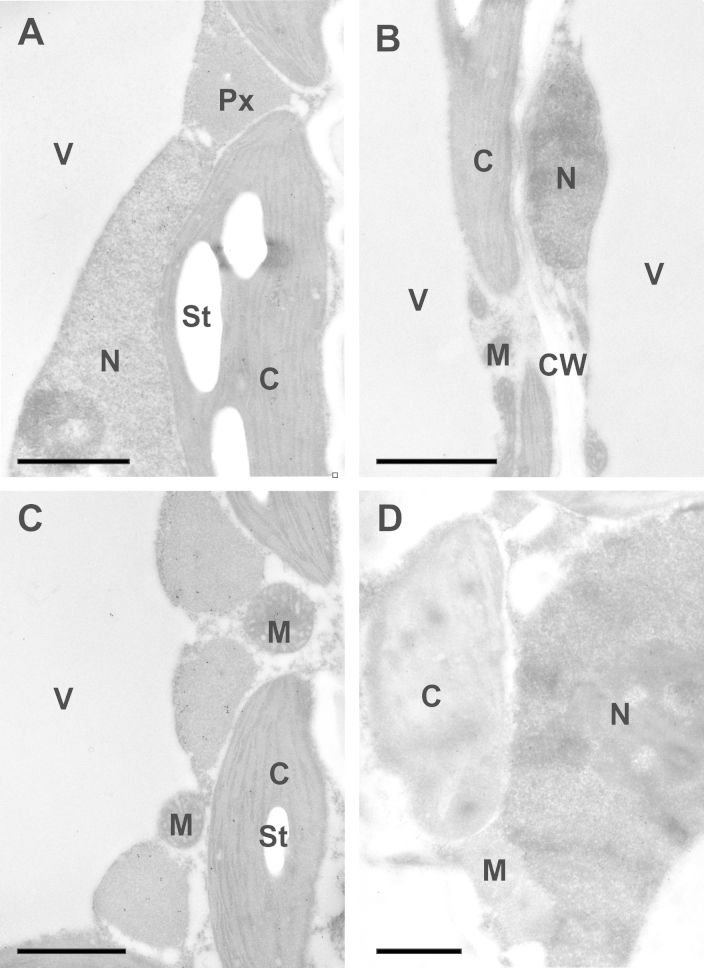


**Figure:**
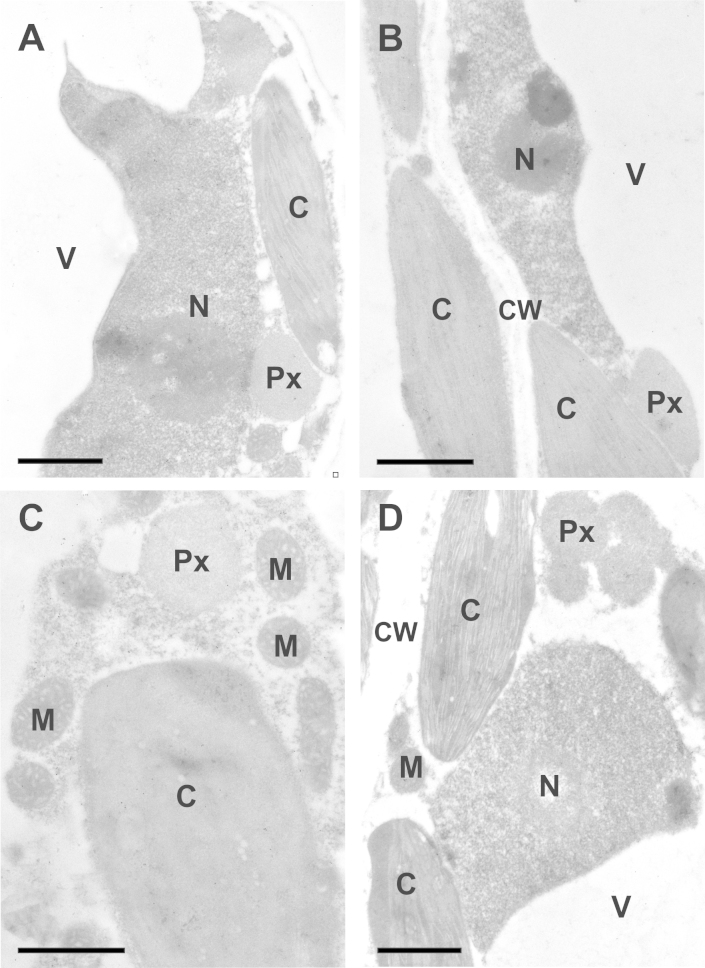


**Figure:**
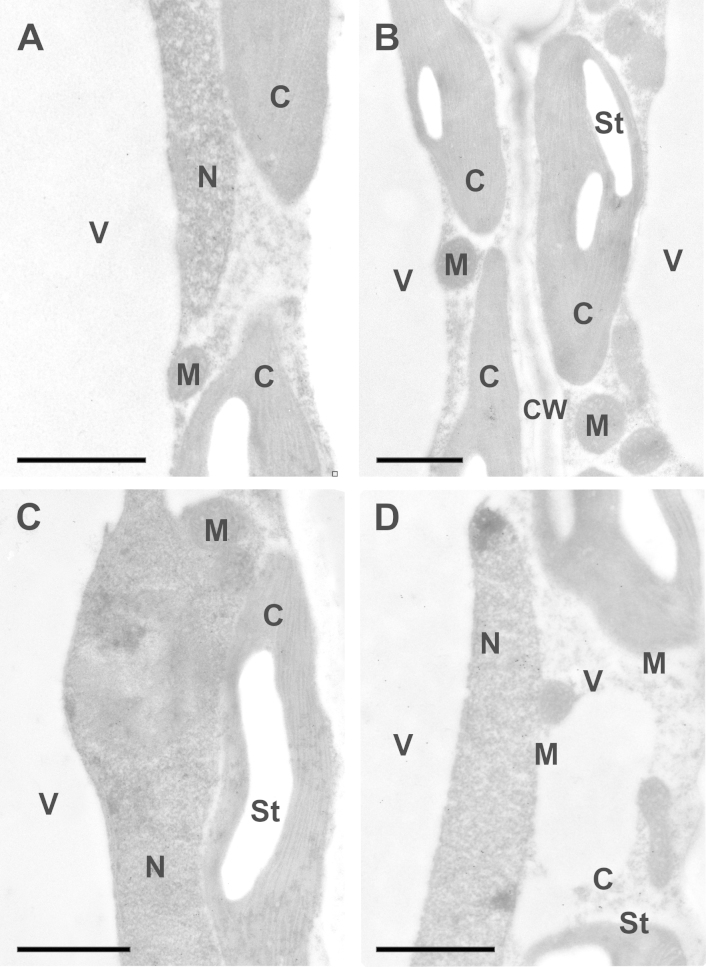


**Figure:**
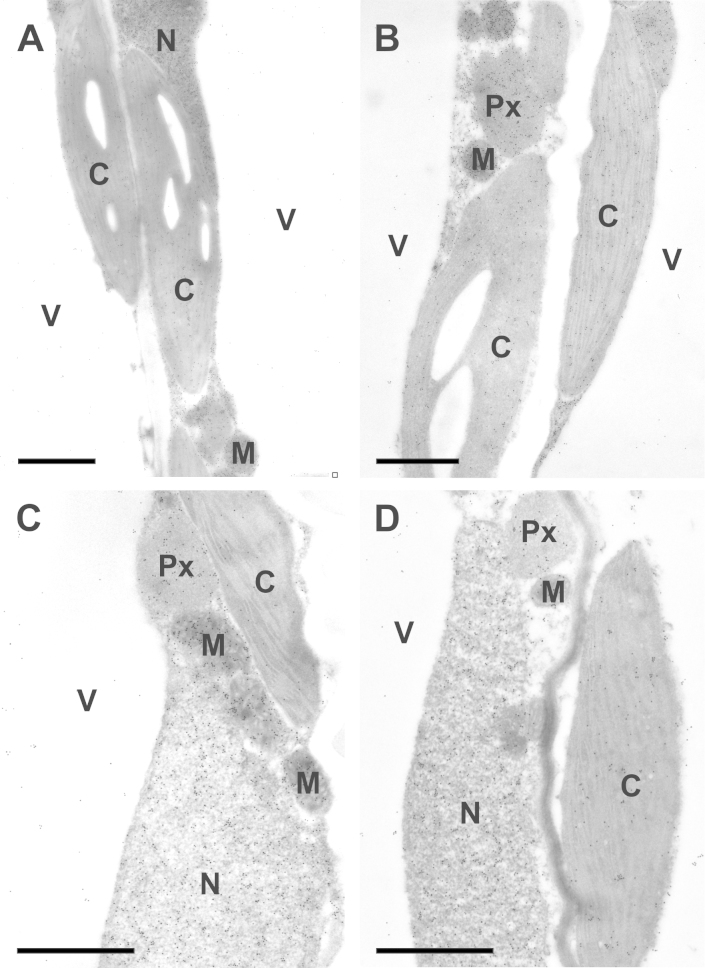


**Figure:**
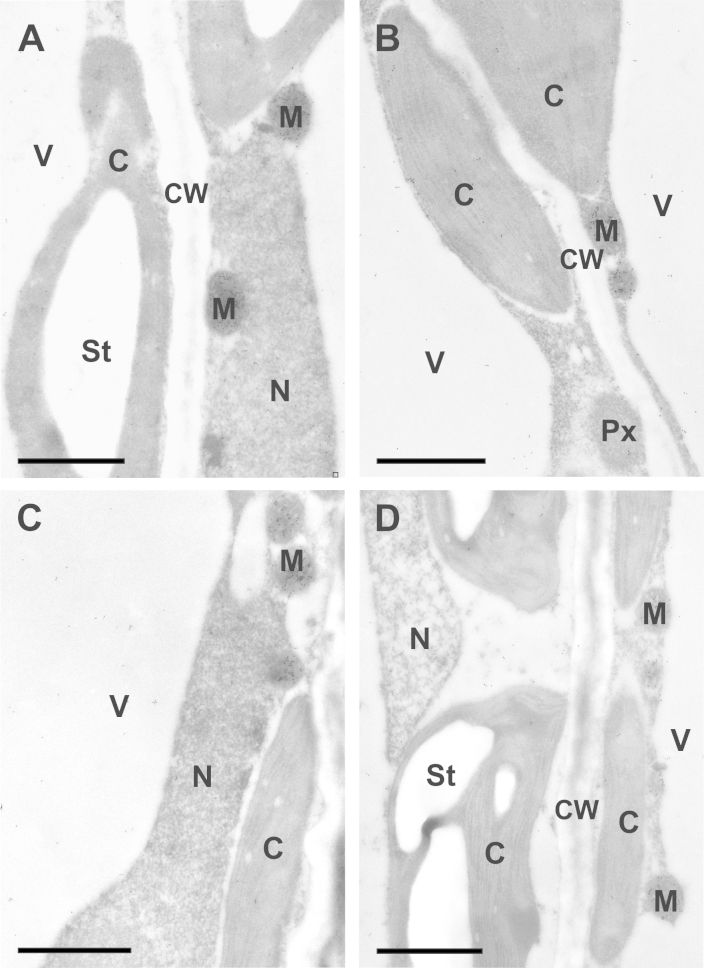


**Figure:**