# Supplementary material for: Compartment specific response of antioxidants to drought stress in Arabidopsis
Source: Plant Sci. 2014 Oct;227:133–44. doi: 10.1016/j.plantsci.2014.08.002 (PMC4180016; doi:10.1016/j.plantsci.2014.08.002)
Supplement: Supplementary file 1 [file mmc1.docx]

|  | Col-0 | *pad2-1* | *vtc2-1* |
| --- | --- | --- | --- |
| Mitochondria | 6.9 ± 1^ef^ | 6 ± 1^fg^ | 1.3 ± 0.5^i^ |
| Chloroplasts | 5.2 ± 0.5^g^ | 8.3 ± 0.6^d^ | 3.4 ± 0.3^h^ |
| Nuclei | 15.1 ± 1^a^ | 11.4 ± 1^b^ | 8.3 ± 0.8^d^ |
| Peroxisomes | 7.8 ± 0.7^de^ | 9.9 ± 1^bc^ | 4.9 ± 0.6^g^ |
| Cytosol | 10.4 ± 1^b^ | 8.6 ± 0.8^cd^ | 2.6 ± 0.7^h^ |
| Vacuoles | 0.8 ± 0.1^ij^ | 1.4 ± 0.1^i^ | 0.4 ± 0.7^j^ |

Supplementary Table A. 1: Values are means with standard errors and document the total amount of gold particles bound to ascorbate per µm^2^ in different cell compartments of *Arabidopsis thaliana* [L.] Heynh. ecotype Columbia (Col-0), *pad2-1* and *vtc2-1* mutants grown at control conditions. n > 20 for peroxisomes and vacuoles and n > 60 for other cell structures. Data are means with standard errors. Different lowercase letters indicate significant differences (P<0.05) analyzed with the Kruskal-Wallis test followed by post-hoc comparison according to Conover.

|  | Col-0 | *pad2-1* | *vtc2-1* |
| --- | --- | --- | --- |
| Mitochondria | 550 ± 26^b^ | 630 ± 16^a^ | 521 ± 24^b^ |
| Chloroplasts | 59 ± 2^h^ | 12 ± 0.7^k^ | 93 ± 6^g^ |
| Nuclei | 391 ± 23^d^ | 28 ± 2^i^ | 469 ± 61^c^ |
| Peroxisomes | 258 ± 7^e^ | 20 ± 3.8 ^j^ | 383 ± 44^d^ |
| Cytosol | 181 ± 10^f^ | 25 ± 1.6 ^ij^ | 188 ± 30^g^ |
| Vacuoles | n.d. | n.d. | n.d. |

Supplementary Table A. 2: Values are means with standard errors and document the total amount of gold particles bound to glutathione per µm^2^ in different cell compartments of *Arabidopsis thaliana* [L.] Heynh. ecotype Columbia (Col-0), *pad2-1* and *vtc2-1* mutants grown at well watered control conditions. n > 20 for peroxisomes and vacuoles and n > 60 for other cell structures. n.d.=not detected. Data are means with standard errors. Different lowercase letters indicate significant differences (P<0.05) analyzed with the Kruskal-Wallis test followed by post-hoc comparison according to Conover.

| mg/g fresh weight | Col-0 | *pad2-1* | *vtc2-1* |
| --- | --- | --- | --- |
| GR | 19 ± 2 | 15 ± 1 | 19 ± 3 |
| DHAR | 85 ± 15 | 60 ± 7* | 25 ± 3** |
| APX | 96 ± 18 | 82 ± 7 | 115 ± 13 |

Supplementary Table A. 3: Values are means with standard errors and document the activity (in nkat/g fresh weight) of glutathione reductase (GR), dehydroascorbate reductase (DHAR), and ascorbate peroxidase (APX) in leaves of *Arabidopsis thaliana* [L.] Heynh. ecotype Columbia (Col-0), *pad2-1* and *vtc2-1* mutants grown at well watered control conditions. Data are means with standard errors. Significant differences were calculated by using the Mann Whitney U-test; * and **, respectively, indicate significance at the 0.05 and 0.01 levels of confidence. n>9 plants.

| mg/g fresh weight | Col-0 | *pad2-1* | *vtc2-1* |
| --- | --- | --- | --- |
| Chlorophyll a | 13480 ± 2205 | 13503 ± 495 | 12670 ± 2038 |
| Chlorophyll b | 1127 ± 86 | 1291 ± 36 | 1062 ± 68 |
| ß-Carotene | 426 ± 25 | 557 ± 13* | 403 ± 29 |
| Lutein/Zeaxanthin | 685 ± 40 | 1034 ± 27** | 664 ± 42 |
| Neoxanthin | 221 ± 15 | 286 ± 8 | 199 ± 14 |
| Violaxanthin | 111 ± 7 | 81 ± 3* | 133 ± 16 |

Supplementary Table A. 4: Values are means with standard errors and document the amount of pigments (mg/g fresh weight) in leaves of *Arabidopsis thaliana* [L.] Heynh. ecotype Columbia (Col-0), *pad2-1* and *vtc2-1* mutants grown at well watered control conditions. Data are means with standard errors. Significant differences were calculated by using the Mann Whitney U-test; * and ** respectively, indicate significance at the 0.05 and 0.01 levels of confidence. n>6 plants.

|  | Col-0 | *pad2-1* | *vtc2-1* |
| --- | --- | --- | --- |
| Fm/Fv | 0.818 ± 0.001 | 0.819 ± 0.001 | 0.812 ± 0.005 |
| NPQ | 0.355 ± 0.02 | 0.313 ± 0.01 | 0.181 ± 0.02*** |
| A | 2.448 ± 0.08 | 2.949 ± 0.11** | 1.279 ± 0.14*** |
| TR | 1.983 ± 0.1 | 1.904 ± 0.08 | 1.789 ± 0.1 |
| Cond | 0.201 ± 0.01 | 0.207 ± 0.01 | 0.188 ± 0.01* |
| WUE | 1.316 ± 0.006 | 1.607 ± 0.005* | 0.769 ± 0.02*** |

Supplementary Table A. 5: Values are means with standard errors and document chlorophyll fluorescence (Fv/Fm, NPQ), net photosynthesis in µmol CO_2_ m-^2^ s-^1^ (A), stomatal conductance in mol H_2_O m^-2^ s^-1^ (Cond), transpiration rate in mmol H_2_O m^-2^ s^-1^ (TR), and water use efficiency in (WUE determined as net photosynthesis/transpiration rate) in leaves of *Arabidopsis thaliana* [L.] Heynh. ecotype Columbia (Col-0), *pad2-1* and *vtc2-1* mutants grown at well watered control conditions. Significant differences were calculated between control and drought stressed plants by using the Mann Whitney U-test; * and *** respectively, indicate significance at the 0.05 and 0.001 levels of confidence. n>10 plants.
